# Early neuro-rehabilitation in traumatic brain injury: the need for an African perspective

**DOI:** 10.1186/s12916-023-03009-z

**Published:** 2023-08-04

**Authors:** Franklin Chu Buh, Peter J. A. Hutchinson, Fahim Anwar

**Affiliations:** 1 Department of Animal Biology and Conservation, Faculty of Science, University of Buea, P.O. BOX 63, Buea, S.W. Region Cameroon; 2 NIHR Global Health Research Group On Acquired Brain and Spine Injury, Cambridge University, Cambridge, UK; 3 Department of Physiotherapy and Physical Medicine, Faculty of Medicine and Pharmaceutical Sciences, University of Dschang, Dschang, Cameroon; 4 Department of Physiotherapy, St. Louis University Institute, Douala, Cameroon; 5 Panafrican Hospital Center, LT Region, P.O. BOX 13152, Douala, Cameroon; 6 Department of Clinical Neuroscience, University of Cambridge, Cambridge, UK; 7 Department of Rehabilitation Medicine, Addenbrooke’s Hospital, Cambridge University Hospitals NHS Foundation Trust, Cambridge, UK

**Keywords:** Early neurorehabilitation, Traumatic brain injury, African perspective

## Abstract

**Background:**

Traumatic brain injury (TBI) is a global public health challenge, affecting about 69 million individuals annually and being one of the leading causes of mortality. It has adverse consequences in terms of cognitive and physical functioning, which makes rehabilitation interventions an integral part of its management. Early neuro-rehabilitation guidelines for traumatic brain injury have not yet been developed and implemented in most of Africa especially Sub-Saharan Africa.

**Body:**

We aimed with this Opinion to propose a collective reflection on the development and implementation of early neuro-rehabilitation guidelines as an integral part of the care in traumatic brain injury. The different aspects to be considered for reflection have been highlighted: Traumatic brain injury severity to be considered in early neuro-rehabilitation; who should be assessed and receive early neurorehabilitation, barriers to be considered for early neurorehabilitation; what early neurorehabilitation to be considered; the different phases involved in rehabilitation after mild, moderate, and severe TBI; and lastly, what perspective for the creation of neurorehabilitation teams. In conclusion, neuro-rehabilitation should start at the time of admission and should continue from the intensive care unit through the community for the moderate-to-severe traumatic brain injury population. However, mild TBI should also be considered for long-term follow-up in the community due to the fact that some mild traumatic brain injury patients might develop chronic cognitive problems or fatigue with time.

**Conclusion:**

Neurorehabilitation should start at the time of admission and continue from the intensive care unit through the community for the moderate-to-severe traumatic brain injury population. There is a need to develop, agree on, and implement guidelines on early neuro-rehabilitation interventions for patients with moderate to severe traumatic brain injury in the African region, where disparities in care are common reality.

## Background

Traumatic brain injury (TBI) is a global public health challenge, affecting about 69 million individuals annually and being one of the leading causes of mortality [1, 2]. In sub-Saharan Africa (SSA), an estimated 3.2 million people sustain TBI annually, and these numbers are expected to rise to 14 million by 2050 [3]. It is a major cause of disability among young adults, and there is a need, therefore, for multidisciplinary management for a healthier and more productive population [4]. Furthermore, recent studies estimated that over 40% of patients who were hospitalized as a result of an acute moderate to severe traumatic brain injury show long-term disability [5]. Therefore, TBI has adverse consequences in terms of cognitive and physical functioning, which makes rehabilitation interventions an integral part of its management [6]. Meanwhile, these services are effectively implemented in developed countries, but this is often overlooked in most of SSA. Therefore, the objective of this viewpoint is to highlight the importance of early neuro-rehabilitation in the management of traumatic brain injury patients and the increasing need for the development and implementation of early neurorehabilitation guidelines reflecting the African context.

## Management of traumatic brain injury

The management of traumatic brain injury is ideally based on the initial severity of the TBI and any other sustained injuries, how those injuries developed or changed over time, and how the injuries affected the person’s capacity for self-care [7]. For instance, the management of severe TBI is ideally based on protocol-based guidelines provided by the Brain Trauma Foundation, and the aims of its management are prophylaxis and prompt management of intracranial hypertension and secondary brain injury, maintenance of cerebral perfusion pressure, and ensuring adequate oxygen delivery to injured brain tissue [8, 9]. Generally, care of a TBI patient should begin at the site of the injury, with the aim of securing the patient’s airway and maintaining adequate ventilation and circulation. Patients with moderate or severe TBI should be transferred to a tertiary care centre with neurosurgical facilities as soon as possible [9]. This is important as outcomes in TBI patients have been found to be influenced by transport methods, the duration of transit, and whether the responding team is led by a physician or a paramedic, as well as discharge against medical advice (DAMA) [9] (Buh et al*.,*2023; accepted). According to the World Health Organization (WHO) Emergency care system [10], emergency care should be delivered minutes to hours after traumatic brain injury and should consist the following: bystander care, notification and dispatch (communication), pre-hospital care at a local clinic or health centre, transportation and referral to an adequate treatment centre, hospital-based emergency care, and then continued care, in a neurosurgical unit, intensive care unit (ICU), etc. It should be noted that half of the patients who die from TBI do so within the first 2 h after injury, making prehospital assessment and interventions, critical [9]. One of the main TBI management pillars is neurosurgical intervention as patients often harbour intracranial hematomas and the mainstay of treatment for substantial hematomas is surgical evacuation. Neurosurgical means include craniotomy, intracranial pressure monitoring, and ventriculostomy [11]. TBI is extremely heterogeneous; therefore, people who experience traumatic brain injury may follow multiple care pathways and receive multiple types of interventions to assist them in recovering from the physical, cognitive, emotional, and behavioural consequences of their injuries. Care options include inpatient and/or outpatient rehabilitation, nursing home care, and community-based services [7]. Disparities in TBI care between high-income countries (HIC) and low-middle-income countries (LMICs) is a common reality from injury site to after-discharge rehabilitation, as most LMICs do not have sufficient resources to implement safe care after TBI [12]. Furthermore, sub-Saharan African countries have underdeveloped trauma systems. Consistent in the narrative is the rural–urban disparity in trauma care access and the disadvantage of the poor [13]. Buh et al. [14] in Cameroon identified disparities in TBI care provision attributable to financial constraints regarding computed tomography (CT) scanning and continuation of care, since health insurance for all is not available. Furthermore, neurosurgical care which is one of the pillars in the management of traumatic brain injury is not sufficient in Africa. Despite the high burden of TBI in Africa, there is disproportionately low access to neurosurgical services, which are most often only localized in big towns or cities at the detriment of the rural communities which have little or no such services. Barriers to seeking neurosurgical services in Africa are mainly due to socioeconomic factors of cost, lack of infrastructure, and human resources, which continue to be at the heart of poor delivery of health care generally in the region [15]. Also, it is estimated that about five million neurosurgical cases go untreated each year and that Africa has one of the highest neurosurgical workforce deficits [16, 17]. According to Ukachukwu et al. [18] Africa will have 3418 neurosurgeons by 2030, with a deficit of 5191 neurosurgeons, based on population workforce targets. This alone indicates a large disparity that would greatly impact TBI care in Africa. Furthermore, multidisciplinary or neurorehabilitation teams are lacking in most of Africa. These disparities down the chain of TBI care greatly affect rehabilitation outcomes after TBI.

## Rehabilitation and its importance in overall TBI care

Rehabilitation has been defined by the World Health Organization as “a set of interventions designed to optimize functioning and reduce disability in individuals with health conditions in interaction with their environment” [19]. Neurorehabilitation of TBI is a vast, multifaceted topic ranging from early rehabilitation of patients with impaired cognitive, physical, communication, and sensory function resulting from nervous systems problems to support and reintegration of patients in their social and professional environments [5]. The existing research suggests that early onset neurorehabilitation in the trauma centre and more intensive neurorehabilitation in the rehabilitation environment help to aid recovery and improve functional outcomes in patients with moderate to severe TBI as compared to standard care [20]. Similarly, there is evidence that early neurorehabilitation of patients with highly complex needs is cost-effective in the long term [21, 22]. It has been reported that patients with severe traumatic brain injury who received early rehabilitation interventions experienced shorter acute phases, shorter hospital stays, and better performance at hospital discharge [23]. Moreover, children who received early rehabilitation in intensive care units had shorter and more efficient rehabilitation after discharge [24]. Furthermore, several studies have demonstrated that early mobilizations and respiratory physiotherapy reduce patient stay at the reanimation [25, 26]. A Cochrane Review of adults with traumatic brain injury of working age found that intensive rehabilitation appears to lead to earlier gains [27]. Data on the effects of early neurorehabilitation (ICU, hospitalization) in LMICs in general and in Africa in particular is extremely rare. However, the first and only study in South Africa that evaluated the effects of early neurorehabilitation (Physiotherapy) in the management of paediatric traumatic brain injury and concluded that children who received early physiotherapy services (airway clearance: vibrations, tracheal stimulation, positioning, manual cough assist, suctioning; functional treatment: mobilization in and out of bed, strengthening, get education, bed mobility, caregiver education) well tolerated it and had better outcomes. Also, most of the children who received early neurorehabilitation during hospitalization were not recommended for further treatment after discharge [28]. They, however recommended further studies with larger sample to draw adequate conclusions on the role of early neurorehabilitation in TBI outcome. Furthermore, a case study in India was reported by Lalwani et al. [29] on a 23-year-old man who sustained a TBI (diffuse axonal injury), he received rehabilitation at three levels: (i) in the neurosurgery ICU (guidance and counselling of family members, manual positioning in 2 h intervals, manual chest vibrations and percussion, chest proprioceptive neuromuscular facilitation (PNF), end-expiratory pressure, facilitating technique like quick icing and quick stretch); (ii) in the neurosurgery ward (thoracic expansion exercises, bilateral active range of motion exercises both upper and lower limbs, isometric exercises for quadriceps and hamstrings, stretching, out of bed mobilization with wheelchair); and (iii) physiotherapy rehab (functional electric stimulation, more strengthening for the limbs and trunk muscles, verticalization with tilt tables, coordination exercises, gait re-education). They concluded that the TBI survival had a remarkable progress in the physical and functional health of the patient.

It is important to note that, while early neurorehabilitation after TBI is currently well-established in developed countries, it is not the case in Africa and, in particular, SSA.

A well-coordinated multidisciplinary team with input from a physical medicine and rehabilitation (PMR) consultant is required to provide hyperacute rehabilitation following TBI. Some of the goals of early multidisciplinary rehabilitation after TBI are highlighted in Table 1. Hyperacute rehabilitation is managed with regular multidisciplinary team meetings reviewing the patient’s goals and also meeting with the patients and families to help manage expectations, facilitate transitions through stages of recovery, and try to provide a reasonable picture of the recovery process [6, 30]. Some of the early rehabilitation strategies, like practising bed mobility, transferring to a chair, neuromuscular electrical stimulation, and ambulation [31], are all employed by the team to help prevent complications of immobility.

**Table 1:** Multidisciplinary goals of early rehabilitation after moderate to severe traumatic brain injury

No	Goals
1	Serial assessment of consciousness
2	Posture and tone management during the period of acute care with appropriate orthotics, splints, stretching regimes, medications, and focal tone management such as botulinum toxins/nerve blocks
3	Advice and interventions regarding early mobilization
4	Advice regarding sleep problems
5	Managing agitation, dysautonomia, pain, and bladder and bowel functions
6	Serial assessment of post-traumatic amnesia
7	Early assessment of communication (and swallowing, if appropriate) and provision of assistive technology
8	Help with tracheostomy care and the weaning programme
9	Plan for long-term nutrition
10	Prevent any skin breakdown
11	Assessment of ongoing rehabilitation needs, liaising, and transfer to a long-term rehabilitation facility
12	Assessment of mental capacity and facilitating best interest decisions
13	Providing prognosis
14	Family support

Despite growing evidence on the benefits of early neuro-rehabilitation of patients with moderate to severe TBI, it still remains unimplemented in most of Africa, especially Cameroon. As of today, only a few studies have investigated the effect of integrating rehabilitation into acute TBI care, most of which are in high-income countries [27]. However, some studies report a few barriers to early neurorehabilitation in TBI, including concerns about intracranial pressure (ICP), cerebral blood perfusion, and cerebral perfusion pressure, as well as organizational barriers like increased staff financial resources and concerns about the safety of the interventions for patients in the ICU [23]. In order to improve outcomes and reduce the tremendous burden TBI places on Africa, it is still crucial for Africa to lead the discussion on what kind of early neuro-rehabilitation model should be adopted for patients with TBI and at which stages of the care pathway. Most developed countries have early rehabilitation provisions and guidelines for moderate to severe TBI, with little relevance and applicability in low-income countries. There is a desperate need to develop and implement early neuro-rehabilitation guidelines and services for traumatic brain injury in LMICs, where the burden of TBI is most felt. This would reduce hospital lengths of stay and improve long-term outcomes, as previously reported.

## The need for an African perspective on early neurorehabilitation after traumatic brain injury

The following issues may be the focus of a collective reflection in an effort to develop and implement guidelines for neurorehabilitation in TBI, from hyperacute to community settings in Africa:

### Which TBI severity is to be considered for early neurorehabilitation services?

The majority of mild traumatic brain injury patients are not admitted to the hospital, with a very small proportion admitted to neurosurgical wards for Glasgow Coma Scale (GCS) monitoring. These patients can be safely discharged with head injury advice and should be signposted to the community rehabilitation services if needed. Furthermore, several studies have reported long-term cognitive problems, as well as fatigue and headaches, in mild traumatic brain injuries [32, 33]. This is an indication that although mild TBI is not part of early neurorehabilitation after TBI, since most of them are discharged within 6 to 24 h, they still need to be followed up at the community levels to quickly detect and appropriately respond in case of any cognitive or psychiatric signs, which may greatly affect the quality of life.

On the other hand, individuals with moderate to severe TBI are admitted to the neurological critical care units and should be considered for early inpatient neuro-rehabilitation during their acute hospital stay. Moderate and severe TBIs are most likely to leave cognitive or functional sequelae, which may be ameliorated or prevented if adequate and timely neuro-rehabilitation services are implemented [12].

### Who should be assessed and receive early neurorehabilitation interventions?

Immobilization poses serious consequences on the functional and psychological health of patients [34, 35]. Moderate to severe TBI patients often have relatively long periods of immobilization as some spend considerable stay at intensive care units and hospitalizations. The time point in recommending early rehabilitation services in hospitalizations or intensive care units still constitutes a point of diverse opinions among health practitioners, with great disparities between HICs and LMICs. For instance, in the study of Kreitzer et al. [24] in the USA, 98% of respondents (physicians, physical therapists, and senior nurses) from various hospitals recommended early neuro-rehabilitation (bed mobilization, transfers from bed to chair, ambulation) services for severe TBI patients at the reanimation. Contrarily to a prospective cohort study conducted in a central African country (Cameroon) by Buh et al., *2023,* accepted), no early neurorehabilitation services were recommended for moderate to severe TBI patients in hospitals and in intensive care units, even though some severe TBI patients stayed as long as months in the intensive care unit. Furthermore, no neurorehabilitation team exists in any of the hospitals in Cameroon, contrary to the United States of America (USA), the United Kingdom (UK), and Europe, where well-established neurorehabilitation teams are part of the intensive care unit and manage TBI patients from a very early stage. Therefore, every patient with moderate to severe TBI should be assessed by the multidisciplinary team during the acute phase. While the ICP is being monitored and is labile, a regular review by the multidisciplinary team should still happen.

### What barriers should be considered for early neurorehabilitation?

Regarding the health barriers to early rehabilitation for moderate to severe TBI patients reported by Eghbali et al., [23] stabilization of the ICP was thought to be the pre-requisite before the start of rehabilitation was recommended. This is also in line with the reports by Kreitzer et al., [24] where most health practitioners based their decision on the start of early neurorehabilitation services on the normalization of ICP. Others, however, based their suggestions for early neurorehabilitation on the type of therapy to be offered to the patients and the length of time since the injury.

Considering these barriers, it is, therefore, essential to agree on standard guidelines, in an African context, for initiating early neurorehabilitation interventions for patients with moderate and severe TBI, taking into account the available resources. Interestingly, the argument on the normalization of ICP seemed to be the principal prerequisite for beginning early rehabilitation interventions, especially physical therapy, but the argument on the type of physical therapy modality has not been clarified in the literature, which could be considered a point of reflection. For instance, chest physiotherapy for patients with respiratory complications, bed-posture management to prevent contractures and deformity, and neuromuscular electrical stimulation (NMES) to prevent muscle atrophy may be adopted in situations where ICP is unstable, while other modalities like mobilizations and transfers can be delayed until the ICP normalizes. From our observation in a prospective cohort study in Cameroon, some patients with severe TBI may need to stay in the intensive care unit for months; this will severely impair their ability to mobilize and muscle strength later. Electrical stimulation would be beneficial to limit muscle wasting, and soft tissue manipulation (massage) would improve local circulation while reducing the occurrence of bed sores, which can, in turn, prolong hospital stays for these patients. Also, family support by the multidisciplinary team is very important during this period of early neurorehabilitation interventions by the neurorehabilitation team.

### What early neurorehabilitation interventions should be considered?

Early neurorehabilitation interventions in TBI should be considered by the neurorehabilitation team soon after admissions for moderate to severe TBI following the multidisciplinary team meeting. It should consist of caregiver’s education, acute therapy interventions focusing on therapies for joint mobilization, strengthening exercises, ambulation, neuro-muscular stimulation, bed positioning, chest physiotherapy, and chest proprioceptive neuromuscular facilitation. These acute therapies would help to prevent complications linked to immobility and provide better outcomes in the rehabilitation phase after discharge from intensive care units and hospitals [14]. Other early rehabilitative goals are summarized in Table 1.

### What are the neurorehabilitation phases to be considered?

From an African perspective, after moderate to severe traumatic brain injury, neurorehabilitation provision can be considered in three phases (Table 2):

- ◾*Phase I*: the hyperacute phase, which should start in the ICU and continue in the acute wards during the acute hospital stay. In this phase and depending on the assessment of the multidisciplinary team, patients may receive massage, electrical stimulation where ICP is stable or unstable, to ensure proper circulation and prevent muscle wasting and retractions of soft tissues. Meanwhile, as ICP stabilizes, joint mobilizations, positioning, and chest physiotherapy should be complemented. This is to ensure the flexibility of the joints and soft tissues and to prevent stiffness. Moreover, moderate to severe TBI patients often require invasive mechanical ventilation [36], which increases the risk of complications such as respiratory secretion retention. Therefore, it will be interesting to perform chest physiotherapy to ensure airway clearance in these patients.
- ◾*Phase II*: the subacute phase, where the patient is in a neurorehabilitation centre with full multidisciplinary team support to deal with physical, cognitive, communication, and neuro-behavioural impairments resulting from moderate to severe TBI. The needs of the specific rehabilitation services should be determined at discharge by the neurorehabilitation team, and a seamless transfer should occur from phase I to phase II services.
- ◾*Phase III: *post-acute phase, where patients are supported within their communities by specialist multidisciplinary teams with reviews from the secondary care if needed. In recent years, it has become increasingly apparent that after acute and early hospital treatment and rehabilitation after TBI, there is a need for community-based programs with a focus on enabling TBI patients to reintegrate with life as much as possible [37]. This phase is very important, as it concerns the brain-injured patient’s activities of daily living. Here, all severities of TBI are concerned, as chronic cognitive problems and fatigue may resurface in mild TBI patients.

**Table 2:** Recommendations for early neurorehabilitation tailored to African settings

No	Recommendation
1	Setting up transdisciplinary neurorehabilitation teams that could consist of physicians, physiotherapists, psychiatrists, and clinical psychologists. This is extremely important as the implementation of the below recommendations depends on establishing neurorehabilitation teams
2	Assessments of TBI patients by the neurorehabilitation teams at the hyperacute stage (ICU) and acute stage (Neurosurgical ward or hospitalization)
3	In hyperacute care where intracranial pressure (ICP) is unstable: education of family or caregivers, application of electrotherapy through transcutaneous electro-neurostimulation (TENS) of the lower limbs are necessary to ensure proper circulation and prevent muscle wasting and retractions of soft tissues
4	In hyperacute care where intracranial pressure is stabilized: In addition to TENS application, education, regular changing of positions, and airway clearance (manual chest vibrations and percussion), passive joint mobilization, chest proprioceptive neuromuscular facilitation)
5	In acute care (hospitalization or neurosurgical ward): education, thoracic expansion, and thoracic mobilization exercises; active assisted range of motion to active range of motion of the upper and lower limbs; static stretching of the hamstrings, quadriceps; isometric contractions of the quadriceps, gluteal muscles, hamstrings, anterior and posterior lodge muscles of the leg, biceps and triceps; bed mobility; ambulation out of bed with a wheelchair. Psychologic or psychiatric intervention, depending on the patient’s cognitive behaviour
6	In the subacute phase, where patients are discharged to physiotherapy services, or neurorehabilitation centres; depending on their physical ability, the following may be considered: continuation of active range of motion, continuation of strengthening programme for the limbs and trunk with gradually increasing intensity, verticalization using a tilt table, or standing up, coordination exercises, proprioceptive exercises, functional electrical stimulation, integration of neurophysiologic techniques (Bobath, PNF), gait re-education. These exercises must be done bearing in mind that repetition is key to the induction of neuroplasticity
7	Post-acute phase or chronic phase when patients are within their communities. Community-based rehabilitation programs (CBR) should be established and implemented with the primary objective of reintegrating TBI survivors into the community as much as possible. According to the Campbell systematic reviews [38], CBR has shown beneficial effects on physical disabilities in stroke patients, and on mental disabilities in patients with schizophrenia. In SSA, most community rehabilitation programs are implemented by missionary hospitals or rehabilitation centres. We recommend that centres managing TBI who do not yet have CBR collaborate with Mission centres that already run these programs
8	Training more Physiotherapists in neurorehabilitation is needed to spearhead the physical care of heterogeneous and life-threatening neurological disorders like TBI

*CBR* community-based rehabilitation, *TBI* traumatic brain injury, *ICP* intracranial pressure, *SSA* sub-Saharan Africa, *TENS* transcutaneous electro-neurostimulation, *ICU* intensive care unit

It is interesting to note that Anwar et al. [39] proposed a rehabilitation model of care for acquired brain injury rehabilitation in Pakistan (a developing country with limited rehabilitation resources), and a similar model of care could be adopted for Africa, thinking about the resources, demographics, and needs of the African population.

### What perspective on neurorehabilitation teams in the context of limited resources in Africa?

Despite limited resources in the African region, we strongly recommend putting in place neurorehabilitation teams in the level 1 trauma centres, taking care of patients with traumatic brain injury. According to the National Health Service (NHS) [40], a neurorehabilitation team consists of a rehabilitation physician, neuro-physiotherapist, neuro-rehabilitation nurse, clinical neuropsychologist, speech and language therapist, dietitian, and neuro-occupational therapist. Albeit specialties like rehabilitation physicians are rare in most of SSA, the other specialists mentioned above are present in most level trauma centres. Thus, experts need to reflect on how these neuro-rehabilitation teams can be constituted in Africa, taking into consideration its limited resources. For instance, a study by Ramaswamy et al. [41] proposes that the trans-disciplinary rehabilitation teams in LMICs for children and adolescents with mental health disorders view their limited resources. The possibility of developing trans-disciplinary neuro-rehabilitation teams for patients with TBI in Africa, considering their limited resources, could be less cumbersome than setting up multidisciplinary teams. Moreover, the trans-disciplinary model was reported to be the gold standard for neurorehabilitation teams because they entail more integrated service delivery than other teams and represent a more patient-centred approach [42].

## Conclusions

In conclusion, neurorehabilitation should start at the time of admission and continue from the intensive care unit through the community for the moderate-to-severe TBI population. There is a need to develop, agree on, and implement guidelines on early neuro-rehabilitation interventions for patients with moderate to severe traumatic brain injury in the African region. This will reduce inequalities in recommending rehabilitation services and the timing of various rehabilitation interventions. Most importantly, it will help TBI survivors by improving their outcomes, which will reduce the burden of TBI in LMICs, where it is quite high, and disparities in care for various reasons are a common reality.

## Data Availability

Not applicable.

## Abbreviations

CT: Computed tomography
DAMA: Discharge against medical advice
GCS: Glasgow Coma Scale
HICs: High-income countries
ICU: Intensive care unit
ICP: Intracranial pressure
LMICs: Low-middle-income countries
NHS: National Health Service
NMES: Neuromuscular electrical stimulation
PMR: Physical medicine and rehabilitation
SSA: Sub-Saharan Africa
TBI: Traumatic brain injury
UK: United Kingdom
USA: United States of America
